# 

**Published:** 1852-06

**Authors:** 


					﻿INDEX TO VOLUME EIGHTH.
\* For original communications, look for the name of the authors as well as the titles
of the subjects. For selected articles look for the titles of the subjects.
For notices of books look for names of authors.
A.	Page.
Anaesthesia, Discussion of, by Phila-
delpha County Med. Society,........	31
Association, American Medical, Pro-
ceedings of the fifth annual meeting
of,..................................  46
Association, American Medical, Com-
mittees for 1852,.........125,	400, 464
Arsenic, Case of Poisoning by, Com-
municated by A. M. Leonard, M. D., 68
Ammonia, Muriate of, in Cynanche
Tonsillaris, by A, B. McKay, M. D., 75
Alvine Discharges in Children,....... 108
American Med. Society in Paris,...... 127
Abdominal Tumor containing hair and
teeth, by T. R. Huntington, M. D.,. 161
Auscultation, Obstetrical, by M. M.
Rodgers, M. D.,................... 301
Association of Southern Central New
York, Transactions of,............ 396
Association of the State of Missouri,
Transactions of,...................397
Association of the State of Illinois,
Transactions of,...................397
Association of the State of Georgia,
Transactions of,.................. 397
Ansemia of Pregnant Females,........501
Aneurism of the A orta, Physical signs
of, by Austin W. Nichols,......... 522
American Jour, of Arts and Sciences,. 524
American Scientific Labors, Encour-
agement of, by Editor,............ 582
Ammonia, Effects of accidental admin-
istration of, by J. H. Beech, M. D.,. 589
Abstinence for three months,........ 618
Adhesive Plaster as a means of Exten-
sion in Fractures, by Prof. Gross,... 622
Page.
Armour, Prof. S. G., on use of purga-
tives in bilious affections of South
and West............................. 697
Anatomical Bill,..................... 714
Arsenical Confectionery,............. 754
Association, American Medical, Trans-
actions for 1852,.................. 756
Addresses, Introductory, etc.,........765
Association, Medical, of Alabama,— 773
Anatomical Bill,..................... 774
B.
Barrett, S., M. D., on Rupture of Ten-
don Achilles,........................ 5
Beech, J. H., M. D., Case of Disloca-
ted Femur reduced by Dr. Reid’s
method,............................. 71
Beech, J. H., M. D., on Bandage after
Deliery,............................237
Bebeerine, sulphate of, by Prof. Patter-
son, .............................. 110
Bandage, Use of, after Delivery,. .112, 237
Bernard’s Physiological Researches, by
J. C. Dalton, Jr., M. D.,....... 129, 193
Buffalo Medical College, session of,
1852-3,...................... 179,	460
Buffalo Medical College, curators of,.. 254
Buffalo Medical College,............. 715
Bartlett on Fevers, third edition,... 186
Biddle’s Materia Medica,............. 187
Boardman, J., A. M., Supplement to
Fracture Tables of Prof. Hamilton,. 203
Buffalo Hospital of the Sisters of Char-
ity,.............................. 25f
Buffalo Hospital of the Sisters of Char-
ity,............................... 776
Page.
Buffalo, Health of, by Editor,......252
Beech, J. H., on Paracentesis in Em-
pyema,............................. 355
Beech, J. H., on effects of administra-
tion of ammonia,................. 589
Bailey, S. G., M. D., on tumor in uterus, 489
Bell, John, M. D., new work by,.....526
Blood changes in disease,........... 578
Bond’s Dental Surgery,.............. 587
Bright’s Disease, review of Dr. French’s
work,.............................. 641
Bright’s Disease,................... 692
Burwell, Dr. Geo. N., on Chloroform
in Midwifery,.................... 775
C.
Conicine, by Orfila, translated by C.
A. Lee, M. D....................... 1
California, Hospitals, Diseases, etc.,.. 104
Cupping, dry, by Dr. Washington,— 116
Chloroform in Obstruction of Bowels,
by Dr. Cain,..................... 118
Chloroform, death while under the in-
fluence of,......................... 176
Chloroform, Poisonous, by Dr. C. T.
Jackson,......................... 381
Chloroform, Thoughts on, by Prof. Gil-
man,............................. 374
Cooper, Bransby, Lectures on Surgery, 184
Carnochan’s Amputation of Lower
Jaw,............................. 189
Cholera, Epidemic, Exciting cause of,
by Prof. F. H. Hamilton,......223, 304
Cod Liver Oil, External use of,.....245
Cholera at Buffalo, by Editor,.......332
Carbuncle, Case of, by Clinton Cole-
grove, M. D.,.................... 357
Colegrove, Clinton, M.D., Case of Car-
buncle,............................ 357
Consumption, Influence of Climate
on,.............................. 389
Consumption, by	Dr. Burnet,......... 428
Chabert on Cholera,................. 398
Convulsions from eccentric causes, by
S. B. Hunt, M. D.,............... 418
Cholera, Pathology of, etc., by J. Law-
rence Paige, M. D.,...............425
Colloquia de Omnibus	Rebus,........435
Chloroform, Death ascribed to, cases
in the Mass. Gen. Hospital,...... 509
Cupping Instrument, by S. C. Rogers,
M. D.,........................... 526
Callus,Provisional, by Prof. F. H. Ham-
ilton, .......................... 529
Castor Bean Epidemic,............... 564
Cholera in Rochester,............... 570
Copperhead, Bite of, treated with
whisky,.......................... 703
Concours in France, abolition of,... 719
Chrono-thermal Med. School,..........720
Croup, Lecture on, by M. Trousseau,. 740
Cholera,............................ 755
Clinical Phrase Book,................774
D.	Page.
Dislocation of Femur left unreduced,.	6
Dislocation of Femur reduced by Dr.
Reid’s method, by J. H. Beech, M. D., 71
Dislocation of Femur into Perineum,
by Professors Parker <fc Pope,...... 115
Dislocations, Reduction of, without
Pulleys, by Dr. John Watson....... 585
Drunkenness, Speedy cure of,........ 119
Dalton, Prof. J. C., Jr., on Physiologi-
cal Researches of Bernard,.......129, 193
Dalton, Prof. J. C„ Jr., Report of two
cases of Irritant Poisoning,........ 597
Dalton, Prof. J. C., Jr., on Transforma-
tion of Encysted Entozoa into Tenia, 542
Dalton, Prof. J. C., Jr., Case of Metas-
tatic Abscess,.......................549
Dalton, Prof. J. C., Jr., Review of Dr.
Frerichs’ work on Bright’s Disease, 641
Dowler’s Tableau of New Orleans,— 190
Dysentery, Mercurials in, by S. B.
Hunt, M. D.,....................327, 232
Dysentery, Pathology and Treatment
of, by Editor,.......................246
Dysentery, Malignant, by E. R. Max-
son, M. D.,..........................354
Dysentery, Treatment of,—371, 373, 579
Dysentery, Treatment of, by Prof.
Yandell,.............................463
Diabetes Mellitus, Treatment of, by
Trousseau,...........................452
Drake, Daniel, M. D., Obituary,.....457
Dalton, Prof. J.C., Case of Pneumonia
with Albuminaria,....................613
Diabetes, Mellitus, Contributions to
Chemical and Physiological History
of, by J. Bryant Smith, M. D.,.......657
Doctor, Country,.................... 683
Drake, Daniel, M. D., Discourse on
Life, Character, and Services of, by
Prof. Gross,........................ 717
Drake, Daniel, M. D., Posthumous vol-
ume of work or Interior Valley,— 719
Dislocation of os Humeri, reduced after
five weeks, by Prof. Eve,........... 747
Dysentery, Treatment of, by Dr. Evans 755
Delinquent Subscribers,............. 772
E.
Erysipelas, Treatment of, by Muriated
Tincture of Iron, by Hamilton Bell,
M. D„............................... 174
Essay, Prize, by Editor,.............257
Empyema treated by Paracentesis, by
J. H. Beech. M. D.,................355
Ethics, Medical,.....................369
Epilepsy, Treatment of, by Prof. Hor-
ace Green,.......................... 749
F.
Fever, Continued, Third Clinical Re-
port on, by Editor,.......6, 76, 145, 149
Fever, Infantile and Hydrocephalus,
Differential diagnosis,............. 106
Page.
Fever, Intermittent, treated by a Tere-
binthinate Liniment along the spine, 509
Fever, Continued, Abortive treatment
by Quinia, Remarks on, by Editor,. 517
Flint, Austin, M. D., Third Clinical
Report,...................6,	76, 145, 149
Flint, Austin, M. D., on Pathology and
Treatment of Dysentery,..............246
Flint, Austin, M. D., Prize Essay on
Variations of Pitch, etc.,...........257
Flint, Austin, M. D., Clinical Report
on Chronic Pleurisy,.......337, 401, 465
Fractures, Report on Treatment of, at
Hospital of Sisters of Charity, by
Prof. Hamilton,...................... 65
Fracture Tables, by Prof. F. H. Ham-
ilton, ............................. 203
Fracture of Cranium, Case of, by Jas.
M. Newman, M. D.,................ 733
Flavors of Fruits and Flowers, Artifi-
cial Production of,............... 240
Fever, Yellow, at Charleston, S. C.,_460
French Weights and Measures..........461
Fusel Oil,...........................527
Fever, Intermitting, at Chelsea, Mass., 627
Fever, Intermitting, Quinine and Opi-
um in cold stage of,...............637
Fracture, ununited, cured by subcuta-
neous perforation. By Harvey Jew-
ett, M. D........................... 738
G.
Graham’s Elements of Chemistry,. — 187
Gross ou Urinary Organs, Opinion of
British and Foreign Medico-Chirur-
gical Review,....................242,	253
Gin, Holland, as a medicine,.........388
Gutta Percha, Solution of, in Chloro-
form, by Prof. Dugas,............... 504
Gross, Prof., on Adhesive Plaster as a
means of Extension in Fractures,.. 622
Gross, Prof., Operation of Castration
in Hermaphrodism,................... 629
Glossitis, Four cases of, by T. D.
Strong, M. D.,.................... 667
Graves, Dr., Death of,.............. 763
H.
Hamilton, Prof. F. H., on Unreduced
Dislocation of Femur,................. 6
Hamilton, Prof. F. H., Report on Treat-
ment of Fractures at Hospital of the
Sisters of Charity................... 65
Hydrocephalus and Infantile Fever,
Differential diagnosis of,.......... 106
Hamilton, Prof. F. H., on Painful Tu-
bercle, ............................ 143
Hamilton, Prof. F. H., on Epidemic
Cholera,......................... 223
Hamilton, Prof. F. H., on Upturning
of Soil as cause of Cholera,.......304
Hamilton, Prof. F. H., on Provisional
Callus,.......................... 529
Page.
Hamilton, Prof., New Views on Pro-
visional Callus,................... 772
Huntington, J. R., M. D., on Abdomi-
nal Tumor containing hair and teeth, 161
Hunt, S. B., on Mercurials in Dysen-
tery, ..........................232,	327
Hun£ S. B., on Convulsions..........418
Hunt, S. B., on Registration of Births,
etc.,.............................. 486
Hooping Cough, Treatment of,.......504
Hernia, Reducible, Cure of, by Injec-
tions, ............................. 506
Hermaphrodism, Case of, and Castra-
tion, by Prof. Gross,............... 629
Hats and Baldness, by Editor,.......649
Hall, Marshall, M. D.,............. 718
Horner, Prof., Decease of,..........719
Hats and Baldness, by Subscriber,___739
Hunt, Dr. S. B., Appointment,.......775
I.
Intestine, Perforation of, by E. R.
Maxson, M. D.,.......................202
Idiocy in Massachusetts,........... 456
Iodide of Potassium a remedy for the
affections caused by lead and mer-
cury, ............................. 753
J.
Journals, Medical,................. 651
Jewett, Harvey, M. D., on Tobacco as
a cause of Sciatica,................ 721
Jewett, Han ey, M. D., case of Frac-
ture cured by subcutaneous perfora-
tion,............................... 738
K.
Kousso for Tape-Worm,.............. 171
Kennedy’s Practical Chemistry,......394
Kentucky State Medical Society,.... 450
Kentucky State Med. Society, Trans-
actions of,........................ 759
L.
Lee, Prof. Chas. A., Translation of Or-
fila on Conicine,.................... 1
Lee, Prof. Chas. A., Case of Acute
Atrophy of Liver,....................593
Leonard, A. M., M. D., Case of Poison-
ing by Arsenic,...................... 68
Lactation, Reproduction of,.......... 113
Laryngitis, Acute, Case of, by Prof.
Crosby,............................ 175
Leucocythtemia, by Prof. Wood,...... 178
Leucorrhcea, Memoir on, by W. Tyler
Smith, M. D.,.................... 365
Laryngitis, Case of,............... 383
Laryngitis, bv Wm. H. White, M. D., 487
Lupus cured by Cod Liver Oil,...... 389
London Museums,.................... 638
London, Medical Life in,............390
Livers of Birds, Experiments on,....443
Lead, Nitrate of, External use of,.... 508
Page.
Liver and its Diseases, by W. B. Her-
rick, M. D„......................... 493
Liver, Acute Atrophy of, Case report-
ed by Prof. Chas. A. Lee,......... 593
Lead, White, shall its use be forbidden
as a paint by Public Authority?___632
Lead affections treated by Iodide of
Potassium,........................ 750
M.
Malgaigne’s Operative Surgery, Notice
of,................................ 64
Maclise’s Surgical Anatomy, Notice
of,................................ 64
Mackay, A. B., on Muriate of Ammo-
nia in Cynache Tonsillaris,........ 75
Medical Reform,......................122
Mitchell, S., M. D., on Retention and
Development of Placenta after Dis-
charge of Fcetus,................. 150
Microscopes, Spencer’s,............. 177
Miller’s Principles of Surgery,...... 185
Meigs’ Obstetrics,.................. 185
Maxson, Edwin R., M. D., on Perfora-
tion of Intestine,.................202
Maxson, Edwin R., M. D., on Malig-
nant Dysentery,................... 354
Maxson, Edwin R., M. D., on the Treat-
ment of Rheumatism,............... 612
Mott, Prof., Foreign honor conferred
on,................................399
Medicines administered on tongue,____445
Microscope Binocular,............448,	634
Menstruation Vicarious,............  449
Metastatic Abscess, Case of, by J. C.
Dalton, Jr., M. D.,............... 549
Miller, Prof. Henry, Report on Obstet-
rics,..............................670
Massie’s Eclectic Southern Practice,.. 696
Med. Profession and African Slavery,. 704
Medical Life, Lights and Shadows
of,............................... 770
Med. College of Buffalo, Commence-
ment,............................. 771
Man, what is he?.................... 773
Meteorological	Phenomenon,...........776
N.
Neuralgia, Treatment of, by Prof.
Rives,............................ 114
New York State Med. Society, Semi-
annual meeting of,................ 191
Nitric Acid in Hooping Cough and
Asthma,............................238
New York Medical Politics,...........462
Nervous System, Remarks on, by Dr.
Taylor,............................496
Nature in Disease,...................556
Nichols, Austin W., Report of two
cases of Perforation of Intestine,... 610
Nichols, Austin W., Report of case of
Aneurism,..........................522
New York Preparatory Med. School,. 655
Page
New Jersey Med. Institute,...........655
New York State Med. Society, Pro-
ceedings of annual meeting of,....... 705
Newman, James M., M. D., Case of
Fracture of Cranium,.............. 733
Necrosis of Femur, etc., by C. D. Rob-
inson, M. D.,..................... 735
O.
Ovariotomy, Table of operations, by
Dr. Atlee,........................ 119
Obstruction of Bowels, Treatment of,. 163
Ovarian Section, Merits and Demerits
of,............................... 241
Opium, by Smelfungus,............... 606
Obstruction of Bowels, Two cases, re-
ported by Dr. Fenner,................ 620
Obstruction of Bowels, Observations
on,.............................   688
Orfila Museum and Prizes,........... 767
Obstetric Catechism,................ 769
P.
Pyroligneous Acid as a Gargle, by
John Evans, M. D,,.................... 63
Pennsylvania Med. College, Appoint-
ments, .............................. 126
Placenta, Retention and Development
of, after Discharge of Fcetus, by S.
Mitchell, M. D„................... 150
Pleurisy, Chronic, Clinical Report on,
by Editor,................337, 401, 465
Prostate, Abscess of, by Prof. James
P. White,.......................... 358
Piper’s Illustrations of Surgery,.... 393
Physician’s Visiting List,...........395
Peaslee, Prof., on Duties of Medical
Men,...............................398
Philadelphia, Medical Life in,.......399
Publishers, Parsimony of,........... 399
Pennsylvania Med. l)ep’t, Report of
Faculty,........................... 454
Pirrie’s Surgery,....................458
Pennsylvania State Society, Transac-
tions of,......................... 463
Precocity in Child, Remarkable case of 497
Poisoning, Irritant, Two cases, report-
ed by Prof. J. C. Dalton,......... 597
Peritonitis, from Perforation, in Typh-
oid Fever, Two cases, reported by
Austin W. Nichols,................... 610
Pneumonia with Albuminuria, by J.
C. Dalton, Jr., M. D.,............ 613
Poisoning from Sugar Plums,......... 621
Pericarditis, Rheumatic and Non-
Rheumatic, by Dr. Ormerod,......... 623
Poisoning by Arsenious Acid, by J.
Bryant Smith, M.D.,................ 730
Pneumonia, by Smelfungus,........... 662
Phlebitis, Portal,.................. 687
Pereiria, Jonathan, M. D., Death of,.. 720
Power, Wm., M. D., Obituary,........ 760
Psychological Journal,.............. 770
Q.	Page.
Quackery and the Newspaper Press,. 396
R.
Rattlesnake, Bite of, by S. W. Wood-
house, M. D.,.......................  72
Rodgers, M. M., M. D., on Obstetrical
Auscultation,....................... 301
Registration of Births, Marriages, and
Deaths, in State of New York, by
S. B. Hunt, M.D.,.................486
Rogers, S. C., M. D., New Cupping In-
strument,........................... 326
Ranking’s Abstract,..................586
Rheumatism, Acute, Treatment of, by
E. R. Maxson, M. D.,............. 612
Robinson, C. D., M. D.. Case of Ne-
crosis of Femur,.....................735
S.
Surgeons, Naval, Rank of,........... 121
Spongio-Piline,..................... 126
Spiritual Writings.................. 172
Swett, on Diseases of the	Chest,.... 180
Stone in Bladder, Arabian mode of
treatment of,........................235
Smelfungus, on arrest of Typhus by
Quinia,............................. 361
Smelfungus, Scripta Diffusa,.........481
Smelfungus, on Opium,................606
Smelfungus, on Pneumonia,........... 662
Snoring prevented by Excision of
Uvula,.............................388
Shellac, Solution of,............... 505
Silver, Nitrate of, Discoloration pro-
duced by topical use of,............ 509
Schnapps, Schiedam Aromatic,........526
Spiritual Rappings,.............592,	625
Syphilis, Inoculation for,...........656
Smith, J. Bryant, M. D., Contributions
to Chemical History of Diabetes
Mellitus,..........................657
Smith, J. Bryant, M. D., Case of Poi-
soning by Arsenious Acid,........... 730
Strong, T. D., M. D., Report of four
cases of Glossitis,................. 667
Scarlet Fever, Dr. Wood’s treatment
of,..................................695
Sprains, Treatment of, by “firing,”.. 756
Spiritual Rappers,..................769
T.
Tendon Achilles, Rupture of, by Mus-
cular Effort, by S. Barrett, M. D.,..	5
Page.
Tubercle, Painful, by Prof. H. Hamil-
ton, ............................ 143
Tansy, Oil of, Poisoning with, by Dr.
J. C. Dalton, Jr.,............... 167
Tansy, by Dr. Ely,................. 170
Tenia, Expulsion of, by Pumpkin
Seeds,........................241,	377
Typhus, Arrest of, by Quinia, by
Smelfungus,...................... 361
Tenia, a new article for,...........369
Tenia, Three cases of, by Prof. Patter-
son, ............................ 378
Tully’s Materia Medica,.........395,	525
Typhus and Typhoid Fevers, Treat-
ment of, by Dr. Todd,.............447
Transylvania Med. Journal,..........462
Tamia, Transformation of Encysted
Entozoa into, by Prof. J. C. Dalton, 542
Typhus and Typhoid Fevers, Quinia
in,.........................;.___581
Typhoid Fever, Treatment of, by
Quinia, by Dr. Hayward,...........587
Tobacco as a cause of Sciatica, by Har-
vey Jewett, M. D.,................721
U.
Urinary Affections, a few Hints in
treating of, by G. Bird, M. D.,......239
Uterus, Cancer of, by Robert Lee,
M. D.,............................242
Upham, on Ship Fever,...............461
Uterus, Tumor in walls of, by S. G.
Bailey, M. D.,................... 489
University of New York,............ 775
V.
Valpeau’s Midwifery,............... 186
Variola and Vaccination, Report on,. 565
Veratrum Viride, by Dr. Norwood,.. 575
Virginia Med. and Surg. Journal,.___774
Vicarious Examination for a Medical
Degree,.......................... 775
W.
Woodhouse, S. W., on Bite of Rattle-
snake, .............................. 72
Wood’s Practice of Medicine,......... 183
White, Prof. James P., Case of Pros-
tatic Abscess,.....................358
White, Wm. H., M. D., Case of Laryn-
gitis,............................487
Webster, Hon. Daniel, Autopsy of,... 635
Wry Neck cured without Cutting  701
Wendell, Benjamin F., Obituary,.... 719
rpiIE SEMI-ANNUAJu MEETING of the Erie County Medical Society, will be held the Sth of June
-L next, nt !'•( A. M.. at the American Hall.
				

